# Crystal structure of (*E*,*E*)-2′,4′-di­hydroxy­aceto­phenone azine di­methyl­formamide disolvate

**DOI:** 10.1107/S2056989016003686

**Published:** 2016-03-08

**Authors:** Wen-Juan Li, Hong-Fei Han

**Affiliations:** a Department of Chemistry, Taiyuan Normal University, Taiyuan 030031, People’s Republic of China

**Keywords:** crystal structure, hydrogen bond, (*E*,*E*)-2′,4′-di­hydroxy­aceto­phenone azine

## Abstract

In the title compound {systematic name: 4,4′-[1,1′-(hydrazinediyl­idene)bis­(ethan-1-yl-1-yl­idene)]bis­(benzene-1,3-diol)}, C_16_H_16_N_2_O_4_·2C_3_H_7_NO, the (*E*,*E*)-2′,4′-di­hydroxy­aceto­phenone azine mol­ecule is centrosymmetric, the mid-point of the N—N bond being located on an inversion centre. All the non-H atoms of the azine mol­ecule are approximately coplanar, the maximum deviation being 0.017 (2) Å. An intra­molecular O—H⋯N hydrogen bond occurs between the azine N atom and the hy­droxy group. In the crystal, azine and di­methyl­formamide solvent mol­ecules are linked by O—H⋯O hydrogen bonds.

## Abstract 2

The approximately planar (*E*,*E*)-2′,4′-di­hydroxy­aceto­phenone azine mol­ecule is located on an inversion centre and linked with di­methyl­formamide solvent mol­ecules *via* O—H⋯O hydrogen bonds.

## Chemical context   

Hydrazones are important compounds due to their possible applications in material and coordination chemistry. Fluorescence properties of hydrazones have been reported (Qin *et al.*, 2009 ▸). Many organometallic compounds containing acyl­hydrazone ligands have also been synthesized for their potential magneto-chemical properties (Guo *et al.*, 2010 ▸). In particular, they have received increasing inter­est for their biological activity as anti­oxidants (Kitaev *et al.*, 1970 ▸), and their anti­microbial (Ramamohan *et al.*, 1995 ▸) and anti­viral properties (El-Tabl *et al.*, 2008 ▸; Rollas & Küçükgüzel, 2007 ▸).
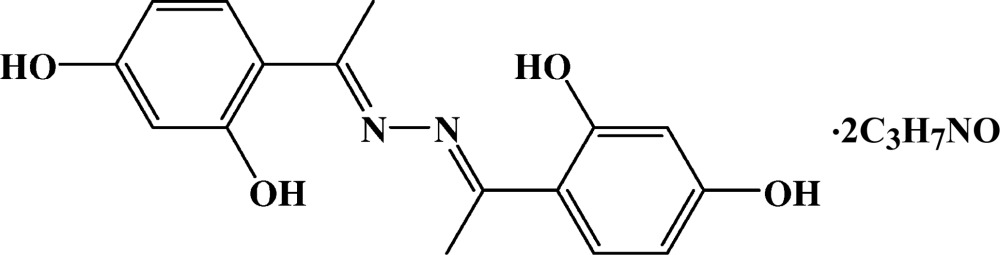


Although 2′,4′-di­hydroxy­aceto­phenone azine has been pre­pared and studied as a fluorescent probe, its structure has not been reported. As a part of our studies on synthesis and structural peculiarities of Schiff base ligands derived from 2′,4′-di­hydroxy­aceto­phenone and hydrazine, we determined the structure of the title compound, (*E*,*E*)-2′,4′-di­hydroxy­aceto­phenone azine di­methyl­formamide disolvate, (I).

## Structural commentary   

**Figure 1:**
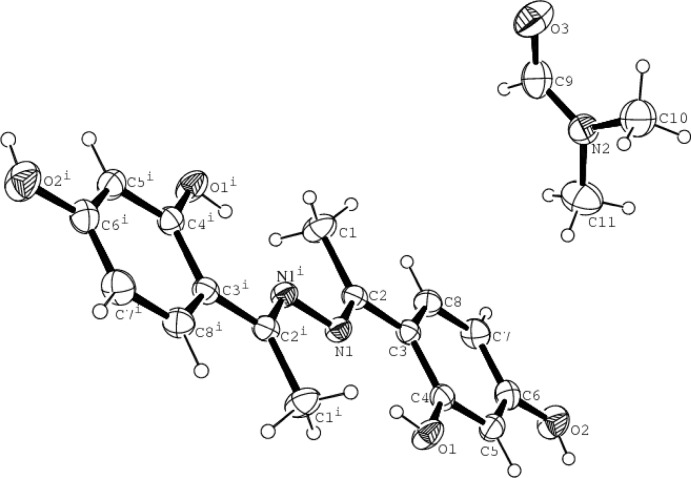
The mol­ecular structure of the title compound, showing the atom-numbering scheme. Displacement ellipsoids are drawn at the 30% probability level. Only one DMF solvent molecule is shown. [Symmetry code: (i) −*x* + 1, −*y* + 1, −*z* + 1.]

The mol­ecular structure of the title compound is depicted in Fig. 1 ▸. The asymmetric unit contains one half-mol­ecule of (*E*,*E*)-2′,4′-di­hydroxy­aceto­phenone azine and one dimethylformamide (DMF) mol­ecule. The complete azine mol­ecule is centrosymmetric and exists in an *E*,*E* configuration with respect to the two C=N bonds. The N1—C2 bond length of 1.301 (3) Å shows double-bond character. The C—O bond lengths [1.349 (3) and 1.358 (3) Å] are comparable with similar bonds in related structures (Chantrapromma *et al.*, 2011 ▸; Tai *et al.*, 2008 ▸). All the non-H atoms of the azine mol­ecule are approximately coplanar. The nine atoms (*i.e.* N1, C1 and C2, and the six C atoms in the benzene ring) are essentially planar, with a mean deviation of 0.0024 Å. Each hy­droxy group is nearly coplanar with its attached benzene ring; the r.m.s. deviation is 0.0045 Å for the seven non-H atoms. Intra­molecular O—H⋯N hydrogen bonds exist in the azine mol­ecule (Table 1 ▸).

## Supra­molecular features   

**Figure 2:**
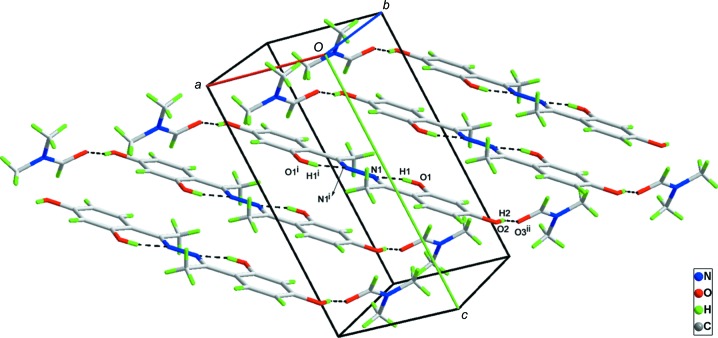
The crystal packing of the title compound. Hydrogen bonds are shown as dashed lines. [Symmetry codes: (i) −*x* + 1, −*y* + 1, −*z* + 1; (ii) *x* − 1, *y* + 1, *z*.]

In the crystal of (I), inter­molecular O—H⋯O hydrogen bonds exist between azine mol­ecules and DMF mol­ecules (Table 1 ▸ and Fig. 2 ▸).

## Database survey   

A search of Cambridge Structural Database (Groom & Allen, 2014 ▸) for aceto­phenone azine gave 105 hits (excluding organometallics). There are four reported crystal structures of aceto­phenone azine containing hy­droxy groups at the 2-position of benzene rings: (*E*,*E*)-2,2′-(1,1′-azinodi­ethyl­idyne)di­phenol (Tai *et al.*, 2008 ▸), (*E*,*E*)-4,4′-di­chloro-2,2′-(1,1′-azinodi­ethyl­idyne)diphenol (Chang *et al.*, 2007 ▸), (*E*,*E*)-3,3′-dieth­oxy-2,2′-(1,1′-azinodi­ethyl­idyne)diphenol (Fayos *et al.*, 1980 ▸) and (*E*,*E*)-4,4′-dimeth­oxy-2,2′-(1,1′-azinodi­ethyl­idyne)diphenol (Zhang *et al.*, 2008 ▸).

## Synthesis and crystallization   

A mixture of 2′,4′-di­hydroxy­aceto­phenone (3.06 g, 20 mmol), hydrazine sulfate (1.28 g, 10 mmol) and tri­ethyl­amine (3.03 g, 30 mmol) in ethanol (40 ml) was heated under reflux for 24 h. After cooling, the precipitate was filtrated and washed with water to afford a yellow solid. Crystals of the title compound suitable for X-ray diffraction were obtained by slow evaporation of a solution of the solid in DMF at room temperature for 5 d (yield 1.20 g, 75%; m.p: 484–485 K). ^1^H NMR (300 MHz, CDCl_3_): δ 13.59 (*s*, 2H, OH), 10.14 (*s*, 2H, OH), 7.58–7.61 (*d*, 2H, ArH), 6.37–6.41 (*d*, 2H, ArH), 6.30–6.31 (*s*, 2H, ArH), 3.34 (*d*, 6H, CH_3_).

## Refinement   

Crystal data, data collection and structure refinement details are summarized in Table 2 ▸. H atoms were placed geometrically (C—H = 0.93–0.96 Å and O—H = 0.82 Å) and refined as riding, with *U*_iso_(H) = 1.2*U*_eq_(C) for aromatic H atoms or 1.5*U*_eq_(C,O) for methyl and hy­droxy groups.

## Supplementary Material

Crystal structure: contains datablock(s) I, global. DOI: 10.1107/S2056989016003686/xu5885sup1.cif

Structure factors: contains datablock(s) I. DOI: 10.1107/S2056989016003686/xu5885Isup2.hkl

Supporting information file. DOI: 10.1107/S2056989016003686/xu5885Isup3.cml

CCDC reference: 1457201

Additional supporting information:  crystallographic information; 3D view; checkCIF report

## Figures and Tables

**Table 1:** Hydrogen-bond geometry (Å, °)

*D*—H⋯*A*	*D*—H	H⋯*A*	*D*⋯*A*	*D*—H⋯*A*
O1—H1⋯N1	0.82	1.82	2.543 (2)	147
O2—H2⋯O3^i^	0.82	1.84	2.649 (3)	171

Symmetry code: (i) 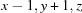.

**Table 2:** Experimental details

Crystal data	
Chemical formula	C_16_H_16_N_2_O_4_·2C_3_H_7_NO
*M*_r_	446.50
Crystal system, space group	Triclinic, *P* 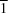
Temperature (K)	298
*a*, *b*, *c* (Å)	6.1616 (7), 7.3109 (8), 13.4537 (15)
α, β, γ (°)	96.771 (1), 103.049 (2), 96.607 (1)
*V* (Å^3^)	579.96 (11)
*Z*	1
Radiation type	Mo *K*α
μ (mm^−1^)	0.09
Crystal size (mm)	0.48 × 0.43 × 0.21

Data collection	
Diffractometer	Bruker SMART CCD area-detector
Absorption correction	Multi-scan (*SADABS*; Sheldrick, 1996 ▸)
*T*_min_, *T*_max_	0.956, 0.981
No. of measured, independent and observed [*I* > 2σ(*I*)] reflections	2902, 2001, 1313
*R*_int_	0.026
(sin θ/λ)_max_ (Å^−1^)	0.595

Refinement	
*R*[*F* ^2^ > 2σ(*F* ^2^)], *wR*(*F* ^2^), *S*	0.055, 0.187, 1.02
No. of reflections	2001
No. of parameters	149
H-atom treatment	H-atom parameters constrained
Δρ_max_, Δρ_min_ (e Å^−3^)	0.28, −0.25

Computer programs: *SMART* and *SAINT* (Bruker, 2007 ▸), *SHELXS97*, *SHELXL97* and *SHELXTL* (Sheldrick, 2008 ▸).

## supplementary crystallographic information

### Crystal data

**Table:**

C_16_H_16_N_2_O_4_·2C_3_H_7_NO	*Z* = 1
*M**_r_* = 446.50	*F*(000) = 238
Triclinic, *P*1	*D*_x_ = 1.278 Mg m^−^^3^
Hall symbol: -P 1	Mo *K*α radiation, λ = 0.71073 Å
*a* = 6.1616 (7) Å	Cell parameters from 1119 reflections
*b* = 7.3109 (8) Å	θ = 3.0–26.5°
*c* = 13.4537 (15) Å	µ = 0.09 mm^−^^1^
α = 96.771 (1)°	*T* = 298 K
β = 103.049 (2)°	Block, colorless
γ = 96.607 (1)°	0.48 × 0.43 × 0.21 mm
*V* = 579.96 (11) Å^3^	

### Data collection

**Table:**

Bruker SMART CCD area-detector diffractometer	2001 independent reflections
Radiation source: fine-focus sealed tube	1313 reflections with *I* > 2σ(*I*)
Graphite monochromator	*R*_int_ = 0.026
phi and ω scans	θ_max_ = 25.0°, θ_min_ = 3.0°
Absorption correction: multi-scan (*SADABS*; Sheldrick, 1996)	*h* = −6→7
*T*_min_ = 0.956, *T*_max_ = 0.981	*k* = −8→7
2902 measured reflections	*l* = −15→15

### Refinement

**Table:**

Refinement on *F*^2^	Secondary atom site location: difference Fourier map
Least-squares matrix: full	Hydrogen site location: inferred from neighbouring sites
*R*[*F*^2^ > 2σ(*F*^2^)] = 0.055	H-atom parameters constrained
*wR*(*F*^2^) = 0.187	*w* = 1/[σ^2^(*F*_o_^2^) + (0.1079*P*)^2^ + 0.0859*P*] where *P* = (*F*_o_^2^ + 2*F*_c_^2^)/3
*S* = 1.02	(Δ/σ)_max_ < 0.001
2001 reflections	Δρ_max_ = 0.28 e Å^−^^3^
149 parameters	Δρ_min_ = −0.25 e Å^−^^3^
0 restraints	Extinction correction: *SHELXL97* (Sheldrick, 2008), Fc^*^=kFc[1+0.001xFc^2^λ^3^/sin(2θ)]^-1/4^
Primary atom site location: structure-invariant direct methods	Extinction coefficient: 0.17 (2)

### Special details

**Table:**

Geometry. All e.s.d.'s (except the e.s.d. in the dihedral angle between two l.s. planes) are estimated using the full covariance matrix. The cell e.s.d.'s are taken into account individually in the estimation of e.s.d.'s in distances, angles and torsion angles; correlations between e.s.d.'s in cell parameters are only used when they are defined by crystal symmetry. An approximate (isotropic) treatment of cell e.s.d.'s is used for estimating e.s.d.'s involving l.s. planes.
Refinement. Refinement of *F*^2^ against ALL reflections. The weighted *R*-factor *wR* and goodness of fit *S* are based on *F*^2^, conventional *R*-factors *R* are based on *F*, with *F* set to zero for negative *F*^2^. The threshold expression of *F*^2^ > σ(*F*^2^) is used only for calculating *R*-factors(gt) *etc*. and is not relevant to the choice of reflections for refinement. *R*-factors based on *F*^2^ are statistically about twice as large as those based on *F*, and *R*- factors based on ALL data will be even larger.

### Fractional atomic coordinates and isotropic or equivalent isotropic displacement parameters (Å^2^)

**Table:**

	*x*	*y*	*z*	*U*_iso_*/*U*_eq_	
N1	0.4279 (3)	0.5237 (2)	0.53085 (13)	0.0391 (5)	
N2	0.5727 (4)	0.1587 (3)	0.91556 (16)	0.0566 (6)	
O1	0.2575 (3)	0.7659 (2)	0.62846 (12)	0.0537 (5)	
H1	0.3372	0.7270	0.5919	0.081*	
O2	−0.2843 (3)	0.5582 (3)	0.80524 (14)	0.0679 (6)	
H2	−0.2793	0.6712	0.8190	0.102*	
O3	0.7469 (4)	−0.0813 (3)	0.87057 (18)	0.0899 (8)	
C1	0.3219 (5)	0.1857 (3)	0.5203 (2)	0.0574 (7)	
H1A	0.2229	0.1462	0.4531	0.086*	
H1B	0.2779	0.1098	0.5681	0.086*	
H1C	0.4737	0.1732	0.5172	0.086*	
C2	0.3080 (3)	0.3858 (3)	0.55569 (15)	0.0374 (6)	
C3	0.1542 (3)	0.4334 (3)	0.62014 (15)	0.0368 (6)	
C4	0.1368 (4)	0.6208 (3)	0.65420 (16)	0.0403 (6)	
C5	−0.0090 (4)	0.6624 (3)	0.71594 (16)	0.0458 (6)	
H5	−0.0180	0.7861	0.7380	0.055*	
C6	−0.1404 (4)	0.5221 (4)	0.74482 (17)	0.0479 (6)	
C7	−0.1270 (4)	0.3382 (4)	0.71238 (18)	0.0528 (7)	
H7	−0.2153	0.2431	0.7316	0.063*	
C8	0.0176 (4)	0.2968 (3)	0.65154 (17)	0.0472 (6)	
H8	0.0249	0.1723	0.6304	0.057*	
C9	0.5795 (5)	−0.0016 (5)	0.8617 (2)	0.0696 (9)	
H9	0.4489	−0.0589	0.8136	0.084*	
C10	0.7669 (5)	0.2551 (5)	0.9909 (2)	0.0808 (9)	
H10A	0.8729	0.1706	1.0084	0.121*	
H10B	0.7228	0.3039	1.0516	0.121*	
H10C	0.8355	0.3556	0.9632	0.121*	
C11	0.3699 (5)	0.2460 (5)	0.8988 (3)	0.0889 (10)	
H11A	0.2480	0.1611	0.8530	0.133*	
H11B	0.3951	0.3568	0.8687	0.133*	
H11C	0.3324	0.2778	0.9635	0.133*	

### Atomic displacement parameters (Å^2^)

**Table:**

	*U*^11^	*U*^22^	*U*^33^	*U*^12^	*U*^13^	*U*^23^
N1	0.0427 (11)	0.0333 (11)	0.0416 (10)	0.0063 (8)	0.0117 (8)	0.0035 (8)
N2	0.0545 (13)	0.0620 (15)	0.0553 (12)	0.0128 (11)	0.0150 (10)	0.0088 (11)
O1	0.0704 (11)	0.0331 (10)	0.0647 (11)	0.0015 (8)	0.0367 (9)	0.0024 (7)
O2	0.0738 (13)	0.0718 (14)	0.0713 (12)	0.0124 (10)	0.0428 (10)	0.0122 (10)
O3	0.0897 (16)	0.0827 (17)	0.1017 (17)	0.0266 (14)	0.0354 (13)	−0.0059 (13)
C1	0.0708 (17)	0.0345 (14)	0.0745 (17)	0.0076 (12)	0.0332 (14)	0.0080 (12)
C2	0.0380 (12)	0.0337 (12)	0.0377 (11)	0.0037 (9)	0.0037 (9)	0.0065 (9)
C3	0.0383 (12)	0.0346 (12)	0.0351 (11)	0.0028 (9)	0.0048 (9)	0.0057 (9)
C4	0.0450 (13)	0.0378 (13)	0.0364 (11)	0.0024 (10)	0.0078 (10)	0.0064 (9)
C5	0.0532 (14)	0.0414 (14)	0.0427 (12)	0.0066 (11)	0.0136 (11)	0.0020 (10)
C6	0.0451 (13)	0.0583 (16)	0.0411 (12)	0.0065 (11)	0.0120 (10)	0.0081 (11)
C7	0.0548 (15)	0.0502 (16)	0.0561 (15)	−0.0022 (12)	0.0199 (12)	0.0155 (11)
C8	0.0539 (14)	0.0385 (14)	0.0497 (13)	0.0031 (11)	0.0137 (11)	0.0102 (10)
C9	0.0675 (19)	0.081 (2)	0.0575 (16)	−0.0021 (16)	0.0183 (14)	0.0061 (15)
C10	0.078 (2)	0.075 (2)	0.078 (2)	0.0069 (17)	0.0043 (17)	0.0000 (16)
C11	0.069 (2)	0.102 (3)	0.102 (2)	0.0268 (19)	0.0191 (18)	0.032 (2)

### Geometric parameters (Å, º)

**Table:**

N1—C2	1.301 (3)	C3—C4	1.417 (3)
N1—N1^i^	1.391 (3)	C4—C5	1.389 (3)
N2—C9	1.313 (4)	C5—C6	1.380 (3)
N2—C10	1.430 (4)	C5—H5	0.9300
N2—C11	1.453 (3)	C6—C7	1.381 (3)
O1—C4	1.349 (3)	C7—C8	1.374 (3)
O1—H1	0.8200	C7—H7	0.9300
O2—C6	1.358 (3)	C8—H8	0.9300
O2—H2	0.8200	C9—H9	0.9300
O3—C9	1.232 (3)	C10—H10A	0.9600
C1—C2	1.503 (3)	C10—H10B	0.9600
C1—H1A	0.9600	C10—H10C	0.9600
C1—H1B	0.9600	C11—H11A	0.9600
C1—H1C	0.9600	C11—H11B	0.9600
C2—C3	1.465 (3)	C11—H11C	0.9600
C3—C8	1.396 (3)		
			
C2—N1—N1^i^	116.3 (2)	O2—C6—C5	122.1 (2)
C9—N2—C10	120.9 (2)	O2—C6—C7	118.0 (2)
C9—N2—C11	121.2 (3)	C5—C6—C7	119.9 (2)
C10—N2—C11	117.9 (3)	C8—C7—C6	119.6 (2)
C4—O1—H1	109.5	C8—C7—H7	120.2
C6—O2—H2	109.5	C6—C7—H7	120.2
C2—C1—H1A	109.5	C7—C8—C3	122.9 (2)
C2—C1—H1B	109.5	C7—C8—H8	118.6
H1A—C1—H1B	109.5	C3—C8—H8	118.6
C2—C1—H1C	109.5	O3—C9—N2	124.5 (3)
H1A—C1—H1C	109.5	O3—C9—H9	117.8
H1B—C1—H1C	109.5	N2—C9—H9	117.8
N1—C2—C3	116.96 (19)	N2—C10—H10A	109.5
N1—C2—C1	122.62 (19)	N2—C10—H10B	109.5
C3—C2—C1	120.4 (2)	H10A—C10—H10B	109.5
C8—C3—C4	116.5 (2)	N2—C10—H10C	109.5
C8—C3—C2	121.9 (2)	H10A—C10—H10C	109.5
C4—C3—C2	121.6 (2)	H10B—C10—H10C	109.5
O1—C4—C5	117.0 (2)	N2—C11—H11A	109.5
O1—C4—C3	122.42 (19)	N2—C11—H11B	109.5
C5—C4—C3	120.6 (2)	H11A—C11—H11B	109.5
C6—C5—C4	120.7 (2)	N2—C11—H11C	109.5
C6—C5—H5	119.7	H11A—C11—H11C	109.5
C4—C5—H5	119.7	H11B—C11—H11C	109.5
			
N1^i^—N1—C2—C3	−179.63 (19)	C3—C4—C5—C6	−0.3 (3)
N1^i^—N1—C2—C1	−0.1 (3)	C4—C5—C6—O2	179.9 (2)
N1—C2—C3—C8	−179.94 (18)	C4—C5—C6—C7	0.2 (3)
C1—C2—C3—C8	0.5 (3)	O2—C6—C7—C8	−179.7 (2)
N1—C2—C3—C4	−0.2 (3)	C5—C6—C7—C8	0.0 (4)
C1—C2—C3—C4	−179.69 (19)	C6—C7—C8—C3	−0.1 (4)
C8—C3—C4—O1	−179.28 (19)	C4—C3—C8—C7	−0.1 (3)
C2—C3—C4—O1	0.9 (3)	C2—C3—C8—C7	179.7 (2)
C8—C3—C4—C5	0.3 (3)	C10—N2—C9—O3	0.1 (4)
C2—C3—C4—C5	−179.47 (19)	C11—N2—C9—O3	178.5 (3)
O1—C4—C5—C6	179.3 (2)		

Symmetry code: (i) −*x*+1, −*y*+1, −*z*+1.

### Hydrogen-bond geometry (Å, º)

**Table:**

*D*—H···*A*	*D*—H	H···*A*	*D*···*A*	*D*—H···*A*
O1—H1···N1	0.82	1.82	2.543 (2)	147
O2—H2···O3^ii^	0.82	1.84	2.649 (3)	171

Symmetry code: (ii) *x*−1, *y*+1, *z*.
